# Risk-Aware Computational Prioritization and Validation Route Design for Medicine–Food Homology Plant Compounds in a Parkinson’s Disease Context

**DOI:** 10.3390/biotech15030066

**Published:** 2026-08-10

**Authors:** Jinhao Zou, Siyi Wang, Jingjiao Yong, Liangyu Yan, Hong Hui, Ye Sun

**Affiliations:** 1 Key Laboratory of Dryness Syndrome in Chinese Medicine, Ministry of Education, Ningxia Medical University, Yinchuan 750004, China; 2 College of Pharmacy, Ningxia Medical University, Yinchuan 750004, China; 3 College of Traditional Chinese Medicine, Ningxia Medical University, Yinchuan 750004, China

**Keywords:** Parkinson’s disease, medicine–food homology, feasibility panel, source provenance, evidence domain assessment, baicalein, molecular docking, experimental validation route, reproducibility

## Abstract

Network pharmacology studies of medicine–food homology plants have identified broad injury response pathways and hubs that cannot support compound-level or Parkinson’s disease (PD)-specific claims. We developed a traceable, non-weighted framework that separates regulatory provenance, PD-context evidence, structural support, and safety/developability liabilities. A ten-plant feasibility panel was locked before overlap with a 1631-gene PD union, yielding 382 plant-associated targets and 190 strict intersections. Leave-one-plant-out analysis retained 173–190 targets, whereas disease source and threshold stress tests showed curation dependence. Whole-blood classifiers showed modest five-fold discrimination (area under the curve, 0.606–0.682) and were excluded from candidate decisions. Redocking-validated AutoDock Vina and protein–ligand interaction fingerprints retained baicalein–MMP9, baicalein–AKT1, and baicalein–BCL2 as caution-tagged follow-up pairs. Quercetin–MMP9 was retained as a liability-tagged comparator, while KCNH2 relations were safety-only. Because no biological validation is presented, these pairs remain hypotheses for prospective MPP^+^-treated SH-SY5Y testing with orthogonal injury, dopaminergic phenotypes, target dependency, material confirmation and safety controls. Baicalein remains source-pending for the material chain.

## 1. Introduction

Parkinson’s disease (PD) is a progressive neurodegenerative disorder characterized by dopaminergic neuronal loss and a broad spectrum of motor and non-motor manifestations. Current clinical management can improve symptoms, but disease modification remains limited, partly because oxidative stress, mitochondrial dysfunction, neuroinflammation and regulated cell death interact across multiple biological levels [1,2,3,4,5,6,7].

Classical pathogenic genes and pathway nodes provide a disease-relevant mechanistic reference frame for computational prioritization in Parkinson’s disease. SNCA, LRRK2, PRKN, PINK1, PARK7, GBA and MAPT are frequently discussed in relation to alpha-synuclein handling, mitochondrial quality control, lysosomal function, oxidative stress, inflammatory activation and neuronal vulnerability [1,2,3]. These mechanisms define the biological modules in which downstream survival, inflammatory remodeling and apoptosis-related targets can be interpreted, but they do not make every downstream node unique to Parkinson’s disease.

1-Methyl-4-phenylpyridinium (MPP^+^) and 1-methyl-4-phenyl-1,2,3,6-tetrahydropyridine (MPTP) models remain widely used to study dopaminergic injury and mitochondrial stress, while SH-SY5Y-based cellular models provide an accessible literature context for interpreting candidate neuroprotective compounds [8,9,10,11]. MPP^+^/MPTP-related models are mechanistically linked to mitochondrial complex I inhibition, oxidative stress, dopaminergic neuronal vulnerability and apoptosis-related signaling.

These oxidative stress, inflammatory, mitochondrial and cell death processes are relevant to medicine–food homology plant research because many plant-derived flavonoids, phenolics, polysaccharides and saponins have been reported to modulate redox balance, inflammatory signaling, mitochondrial homeostasis or programmed cell death. Connecting these disease modules with plant-derived compound–target records can make candidate screening more biologically interpretable than a broad network alone.

Medicine–food homology plants occupy a translationally attractive space between dietary exposure, traditional use and pharmacological investigation. Recent reviews of functional foods and medicine–food homology resources emphasize that plant-derived flavonoids, polysaccharides and saponins may modulate inflammatory, oxidative-stress and neurodegeneration-related processes, but their broad chemical complexity also makes target prioritization difficult [12,13,14,15,16].

For this analysis, the ten plants were treated as a locked feasibility panel drawn from official medicine–food homology records. The lock criteria were regulatory status, clear botanical/material mapping, TCMSP or ETCM retrievability, standardized PubChem/SMILES structures, human target mapping and sufficient chemical diversity for the computational modules. Parkinson’s disease overlap was calculated after the panel was fixed and was not used to admit or exclude a plant. Because the complete official plant pool was not rerun under an exhaustive sampling design, the panel supports a reproducible case analysis but not a comparative claim that these ten plants are superior to all other eligible materials.

Compound-specific PD literature was used only as a downstream evidence domain. Database-level plant–compound links, material-level phytochemical measurements, PD-model literature, tissue or cell localization, structural predictions and chemical liability flags were kept separate. A missing domain was reported as missing rather than converted into a compensating numerical value.

Against this background, the study asked whether a locked medicine–food homology feasibility panel could generate traceable compound–target hypotheses without treating broad pathway overlap, whole-blood classification or docking affinity as mechanism proof. The methodological contribution is a traceable, non-weighted evidence domain framework that separates source provenance, PD-context records, structural quality control evidence and liabilities and applies deterministic downgrade rules. This makes assumptions, missingness and failure modes auditable without converting heterogeneous evidence into an efficacy-like score; it does not demonstrate that a selected compound is effective.

## 2. Materials and Methods

### 2.1. Medicine–Food Homology Plants and Compound–Target Integration

Plant handling followed a two-stage, disease-independent feasibility procedure. Stage 0 defined the regulatory source boundary from the 2002 national list and the 2019, 2023 and 2024 additions [17,18,19,20]. Stages 1–2 assessed official status, botanical/material mapping, TCMSP or ETCM retrievability, PubChem/SMILES structure availability, human target mapping, record completeness and chemical diversity. The resulting ten-plant panel was then locked before PD-associated targets were introduced. It is a reproducible analytical panel, not an exhaustive rank of all official medicine–food homology plants, and no inference is made about plants outside the panel. The procedure is summarized in Table 1. Supplementary Table S1 preserves contemporaneous rule and status records; it is not a retrospectively reconstructed item-by-item exclusion audit. Because the original panel build did not retain a prospective machine-readable exclusion record for every official plant, representativeness against the complete official source pool cannot be reconstructed. The post hoc leave-one-plant-out audit described in Section 2.7 addresses dependence on any single retained plant only; it does not estimate selection bias relative to non-panel materials.

The Stage 0 audit separated official records by material type and mapping status. Animal-derived, mineral-derived and non-plant records were outside the plant analysis. Plant records with unresolved botanical identity, insufficient compound–target retrieval, missing standardizable structures or poor human-target mapping were not forced into the panel. PD target overlap and compound-specific PD literature were not feasibility criteria and were examined only after the panel lock.

Compound–target records were collected from the Traditional Chinese Medicine Systems Pharmacology Database and Analysis Platform (TCMSP; https://tcmsp-e.com/tcmsp.php (accessed on 23 July 2026)) and the Encyclopedia of Traditional Chinese Medicine 2.0 (ETCM2; http://www.tcmip.cn/ETCM2/front/#/ (accessed on 23 July 2026)). Structures were standardized using PubChem compound identifiers (CIDs) or simplified molecular-input line-entry system (SMILES) strings (https://pubchem.ncbi.nlm.nih.gov/ (accessed on 23 July 2026)) [21,22,23]. Baicalein was present in the local TCMSP-derived Polygonati Rhizoma table as MOL002714 and in a *Polygonatum cyrtonema* metabolite table [24], whereas the checked dissertation on P. sibiricum did not identify baicalein [25]. It was therefore retained with source-pending status. No statement treats baicalein as measured in, abundant in or responsible for the effect of a future Polygonati Rhizoma material.

As shown in Table 2, the regulatory source column records the applicable national list or addition. Slash-separated Polygonatum taxa denote the accepted source-species scope for the material record; they do not imply that every species or batch has the same constituent profile. Database plant–compound relations are not interpreted as quantified phytochemistry.

### 2.2. Disease Target Collection and Strict Target Intersection

PD-associated targets were collected from Open Targets (https://platform.opentargets.org/; accessed on 23 July 2026), the Comparative Toxicogenomics Database (https://ctdbase.org/; accessed on 23 July 2026) and Harmonizome (https://maayanlab.cloud/Harmonizome/; accessed on 23 July 2026). Open Targets records were retained at an association score of ≥0.1, yielding 1237 unique human genes after one duplicate row was collapsed. The retained CTD/Harmonizome branch used a score of ≥1.0 and yielded 517 unique genes. Gene-symbol standardization and deduplication against UniProt (https://www.uniprot.org/; accessed on 23 July 2026) produced a union of 1631 unique disease-associated genes. Direct intersection with the 382 plant targets produced 192 genes. Plant-side records whose target name conversion was labeled mapped_candidate rather than ETCM_gene_symbol, mapped_exact or mapped_name_overlap were then excluded from the strict set. This removed GABRA6 and NOS3 and retained 190 strictly mapped targets, equivalent to 11.65% of the disease union and 49.74% of plant targets [26,27,28,29,30]. The raw and strict records, including the two exclusions, are provided in the reproducibility package. Database links were treated as screening records rather than direct biochemical evidence.

### 2.3. Protein–Protein Interaction Network and Functional Enrichment

The 190 intersected targets were submitted to STRING (https://string-db.org/; accessed on 23 July 2026) with Homo sapiens and a required interaction score of 400. Protein–protein interaction (PPI) centrality ranked network position but did not define disease specificity. Functional enrichment used g: Profiler (https://biit.cs.ut.ee/gprofiler/gost; accessed on 23 July 2026) and was grouped into oxidative stress, neuroinflammation, apoptosis/cell death, neurotransmission, mitochondrial metabolism and protein homeostasis [31,32]. Broad enriched pathways were treated as context only.

### 2.4. Public Transcriptomic Context and Exploratory Whole-Blood Classification

Bulk expression was examined in GSE49126 (peripheral blood mononuclear cells [PBMCs]; 20 controls, 30 PD), GSE42966 (substantia nigra; 6 controls, 9 PD) and GSE99039 (whole blood; 233 controls, 205 idiopathic PD) through the Gene Expression Omnibus (https://www.ncbi.nlm.nih.gov/geo/; accessed on 23 July 2026) [33,34]. GSE49126 and GSE42966 used *p* < 0.05 and |log2FC| ≥ 0.25; GSE99039 used a false discovery rate (FDR) of < 0.05 and |log2FC| ≥ 0.10.

Exploratory GSE99039 classification used all 438 samples and 182 detectable intersected targets in scikit-learn v1.8.0 (https://scikit-learn.org/; accessed on 23 July 2026) [35]. StratifiedKFold used five folds, shuffling and random_state = 20260521. The L1-logistic pipeline used StandardScaler and LogisticRegression (penalty = ‘l1’, solver = ‘liblinear’, C = 1.0, class_weight = ‘balanced’, max_iter = 5000, random_state = 20260521). The random forest used 600 trees, max_features = ‘sqrt’, class_weight = ‘balanced_subsample’ and random_state = 20260521. The linear support vector machine (SVM) pipeline used StandardScaler and SVC (kernel = ‘linear’, probability = True, class_weight = ‘balanced’, random_state = 20260521). ROC AUC was calculated from its decision function rather than probability-calibration output. Cross-validated receiver operating characteristic area under the curve was the performance metric. Fold-wise AUCs and descriptive 95% t intervals across the five folds are retained in the reproducibility package. Separate feature records used LogisticRegressionCV with 15 C values from 10^−3^ to 10^1^, a 1000-tree random forest, and StandardScaler followed by LinearSVC (C = 0.05, L2 penalty, max_iter = 10,000) and recursive feature elimination (RFE) to 30 features with step = 0.1. Feature selection, including the scaling preceding SVM-RFE, was fitted to the full cohort rather than nested within evaluation folds; consequently, feature frequencies and internal performance estimates may be optimistic. No external validation was performed. Whole-blood outputs were excluded from every compound–target decision and were not used as neuronal mechanism or diagnostic evidence. KCNH2/human ether-à-go-go-related gene (hERG) was retained only as a cardiac safety liability signal.

Dopamine- and monoamine-related genes present in the 190-target matrix were recorded as phenotype context. Canonical dopaminergic markers absent from that matrix were not added post hoc, and no marker list was used to promote a candidate target.

Detailed dopaminergic phenotype-context definitions are provided in Supplementary Table S2.

### 2.5. Single-Nucleus Localization, Structural Analysis and Binding Prediction

Human ventral substantia nigra single-nucleus data from GSE329625 were used for cell-type localization and an exploratory donor-level comparison. The processed gene-by-nucleus count matrix and author annotations for CellType, Sample_v2, Disease and nCount_RNA were retained. For localization, each nucleus was transformed as log1p (raw gene count/nCount_RNA × 10,000), and author-annotated control (CTR), Parkinson’s disease (PD) and incidental Lewy body disease (ILBD) nuclei were retained. For the exploratory disease comparison, raw counts were aggregated within donor and cell type and transformed as log1p (sum gene count/sum nCount_RNA × 1,000,000). PD and CTR donor summaries were compared with two-sided Welch tests; ILBD donors were excluded. Benjamini–Hochberg correction was reported both across all tested gene-cell-type combinations and within each cell type. This donor-level analysis was prespecified as exploratory and was not used for target prioritization.

RDKit v2025.09.5 (https://www.rdkit.org/; accessed on 23 July 2026) generated Morgan fingerprints, Tanimoto distances, Murcko scaffolds, Lipinski descriptors, a simple blood–brain barrier (BBB) proxy, pan-assay interference compound (PAINS) filters and Brenk structural alert filters. Uniform manifold approximation and projection (UMAP; umap-learn v0.5.12, https://umap-learn.readthedocs.io/; accessed on 23 July 2026) summarized chemical space [36,37,38].

The contrastive protein–ligand embedding model ConPLex v0.1.12 (https://conplex.readthedocs.io/; accessed on 23 July 2026) used reviewed human UniProt amino acid sequences as protein input and standardized ligand SMILES as molecule input; protein structure coordinates were not supplied to ConPLex. Thirty of 31 candidate pairs had a complete sequence-SMILES input. Isorhamnetin-NCF1 lacked a reviewed sequence mapping in the retained input record and therefore had no ConPLex score. ConPLex outputs were retained as continuous relative records without a universal binary positivity threshold. Structures from the RCSB Protein Data Bank (RCSB PDB; https://www.rcsb.org/; accessed on 23 July 2026) were used only in the separate AutoDock Vina and Protein–Ligand Interaction Fingerprint (ProLIF v2.1.0; https://prolif.readthedocs.io/; accessed on 23 July 2026) structural workflow [39,40,41]. For BCL2, 4IEH/1E9 defined the BH3-binding groove; overlap indicated pocket compatibility only.

Protein structures were reduced to altloc blank/A protein atoms; crystallographic ligand, water, and non-selected heteroatom records were removed. Metal records were retained for MMP9 (Zn) and AKT1 (Mn). Selected MMP9 and AKT1 receptors were converted to rigid Protein Data Bank, Partial Charge (Q), and Atom Type (T) format (PDBQT) with Meeko mk_prepare_receptor.py (https://meeko.readthedocs.io/; accessed on 23 July 2026) using the co-crystal box and allow-bad-residues option; BCL2 used the retained 4IEH Meeko receptor record. Selected MMP9 and AKT1 receptors were converted to rigid Protein Data Bank, Partial Charge (Q), and Atom Type (T) format (PDBQT) with Meeko v0.7.1 (mk_prepare_receptor.py; https://meeko.readthedocs.io/; accessed on 23 July 2026) using the co-crystal box and the allow-bad-residues option; BCL2 used the retained 4IEH Meeko receptor record. The original BCL2 receptor preparation command log and the random seed for the initial baicalein experimental torsion knowledge distance geometry/Merck molecular force field (ETKDG/MMFF)-to-Meeko preparation were also unavailable; these gaps are disclosed in Supplementary Table S6 and data/docking_preparation_audit.csv. Other candidate ligands were prepared from standardized SMILES with RDKit ETKDG version 3 (ETKDGv3; seed 20260521), Universal Force Field (UFF) optimization (600 iterations), and Open Babel v3.1.0 (https://openbabel.org/; accessed on 23 July 2026) for hydrogen addition and Gasteiger charge/PDBQT conversion.Controls used ETKDGv3 (seed 20260630), UFF (1000 iterations), and Open Babel. AutoDock Vina v1.2.5 (https://autodock-vina.readthedocs.io/; accessed on 23 July 2026) used a grid spacing of 0.375 Å. Candidate and reference-control runs used exhaustiveness 12, 10 output modes, and seed 20260521 on the same prepared receptor and grid within each target. The grids were MMP9 2OVX/4MR, center (24.783, 8.439, 50.409) and size (22.000, 25.976, 22.000) Å; AKT1 3MV5/XFE, center (5.133, 3.027, 17.947) and size (22.000, 22.000, 22.000) Å; and BCL2 4IEH/1E9, center (12.200, 25.100, 11.100) and size (28.000, 28.000, 28.000) Å. Co-crystal redocking reused the parameters retained in the original logs rather than the candidate/control seed: MMP9 used center (24.783, 8.439, 50.4085), exhaustiveness 12, and seed −218993885; AKT1 used center (5.1335, 3.027, 17.947), exhaustiveness 12, and seed −1106460419; and BCL2 used center (12.2, 25.1, 11.1), exhaustiveness 16, and seed −2087041463; all requested 10 modes. Redocking was accepted at a heavy-atom root mean square deviation (RMSD) <2.0 Å. SB-3CT, MK-2206, and venetoclax were target-oriented docking controls. Redocking audits pose recovery and grid placement, not binding chemistry or potency; the retained-Zn MMP9 model does not fully represent metal coordination chemistry.

### 2.6. Non-Weighted Evidence Domain Assessment

Candidate pairs were assessed in four non-weighted domains: (1) source provenance and material traceability; (2) PD-context evidence from the model literature and substantia nigra or dopaminergic neuron context; (3) structural support from an available ConPLex record, redocking-validated same-grid docking and ProLIF; and (4) developability or safety liabilities, including the BBB proxy, PAINS and Brenk structural alert filters, excessive lipophilicity and KCNH2/hERG. No additive score, letter grade or efficacy rank was calculated. A primary follow-up pair required a disclosed source status, at least one PD-context record and a QC-passed docking/ProLIF profile. A broad high-frequency compound with major liabilities was assigned as a comparator. Missing a core domain produced reserve status, and Compound–KCNH2/hERG pairs were assigned to the safety-only category. Liabilities could downgrade but never upgrade a pair; whole-blood machine learning was excluded from every decision rule (Table 3). The same descriptor calculations and structural alert filters were applied to every retained compound, while source provenance remained a record-level judgment. Transfer to a new disease, database or plant panel would require the same disclosed rule table and remains externally untested.

### 2.7. Post Hoc Robustness and Decision Ablation Audits

Two post hoc robustness audits were performed from retained machine-readable records without changing the locked panel or candidate rules. For the plant panel jackknife, each of the ten plants was omitted once, the remaining unique plant targets were recomputed, and the strict 190-target intersection was recalculated; retention was expressed against the baseline 190. For disease-source sensitivity, the strict baseline set was re-evaluated using the Open Targets branch alone, the CTD/Harmonizome branch alone, higher Open Targets cutoffs (0.2, 0.3 and 0.5) with CTD/Harmonizome fixed at ≥1.0, higher CTD/Harmonizome cutoffs (1.25, 1.5 and 2.0) with Open Targets fixed at ≥0.1, and selected joint threshold combinations. These are deletion and threshold stress tests on the retained dataset, not a new database search or external validation.

Separately, a rule ablation audit compared four counterfactual readings of the retained outputs: PPI centrality only, docking only, whole-blood feature only and source-blind. The audit asked whether removing each safeguard would change narrative priority for observed cases. Because no independent gold-standard outcome labels were available, it was interpreted as a failure-mode audit rather than a predictive performance benchmark. Calculations and scenario-level records are provided in Supplementary Tables S9 and S10 and the reproducibility package.

### 2.8. Reproducibility and Source Status Control

**Figure 1:**
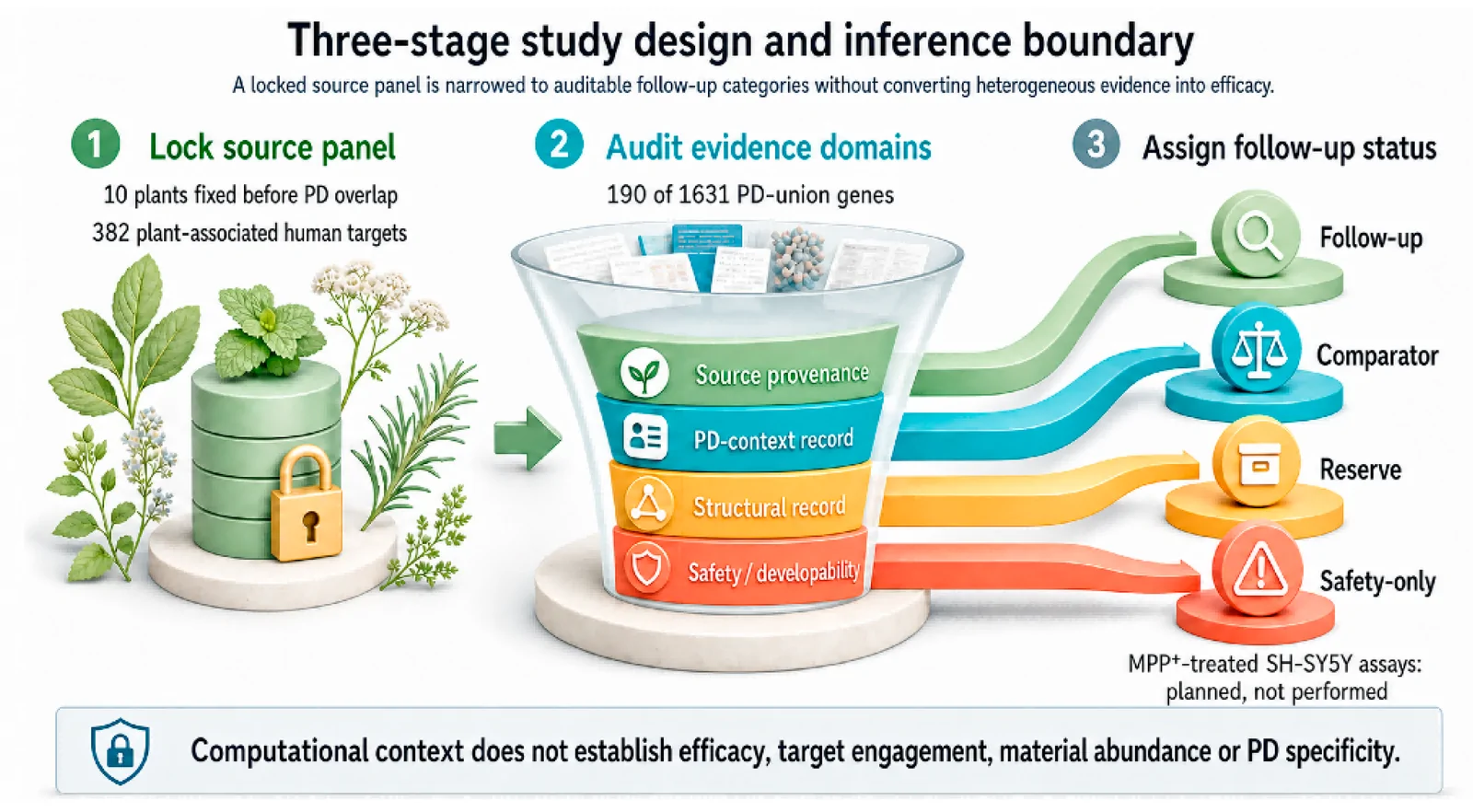
Three-stage study design and inference boundary. The ten-plant feasibility panel was locked before PD-target overlap. Source provenance, PD-context records, structural records, and liabilities were audited as separate domains before categorical follow-up status was assigned. The MPP^+^-treated SH-SY5Y route is planned and was not performed in the present study. Computational context does not establish efficacy, target engagement, material abundance, or PD specificity. Figure 1 was redrawn from an author-defined schematic using an OpenAI generative image tool (OpenAI, San Francisco, CA, USA; accessed 22 July 2026; model identifier not exposed by the interface); all scientific content and labels were specified and verified by the authors. Abbreviations: PD, Parkinson’s disease; MPP^+,^ 1-methyl-4-phenylpyridinium.

Analyses were processed from project-level source tables and intermediate CSV files. Duplicate compound–target records were collapsed at the compound gene level after structure and gene symbol standardization. Supplementary Table S3 provides public URLs, accessions, thresholds and package-relative filenames. The accompanying reproducibility package contains portable derived CSV files, exact GSE99039 model inputs, a five-fold recomputation script, the GSE329625 localization and donor-level count-aggregation outputs with a portable rerun script, the ConPLex sequence-SMILES input and input/prediction audits, selected prepared docking inputs, relative-path Vina configurations, retained outputs, the plant jackknife and disease source/threshold recalculation script, and SHA-256 checksums. Supplementary Tables S4–S7 report dataset and structural records; Supplementary Tables S9–S11 report the robustness, decision ablation and evidence stage comparison audits. Portable rerun scripts were checked under Python v3.12.13 and specify pandas ≥ 2.2, <3, scikit-learn v1.8.0, and NumPy ≥ 2, <3; other analytical packages are versioned in the text or reproducibility package. An OpenAI generative image tool (OpenAI, San Francisco, CA, USA; accessed on 22 July 2026; model identifier not exposed by the interface) was used only to redraw Figure 1 from an author-defined schematic. All labels, numerical values, and inference boundaries were checked manually; no analytical data were generated or altered.

Candidate decisions were derived from the locked computational evidence domains only. Internal consistency requires agreement among disclosed source status, PD-context evidence, structural quality control and explicit liabilities; it does not require every layer to point in the same direction. Missingness and discordance remained visible rather than being averaged away.

Detailed data sources, dataset roles, docking grids and preparation records, reference controls and ProLIF counts are provided in Supplementary Tables S3–S7. Supplementary Table S8 retains the prespecified prospective validation matrix, and Supplementary Tables S9–S11 contain the robustness, rule ablation and evidence stage records.

### 2.9. Prospective MPP^+^-Treated SH-SY5Y Validation Route

Biological validation was not performed in the present study. A prospective MPP^+^-treated SH-SY5Y route was prespecified to test, rather than assume, neuroprotective activity of prioritized compounds. The route begins with cell-line identity and mycoplasma control, an MPP^+^ concentration–time pilot, and compound-only cytotoxicity, precipitation and CCK-8 interference checks. Subsequent independent culture experiments would compare vehicle, MPP^+^ injury, compound alone, MPP^+^ plus a concentration series, a quercetin comparator and an N-acetylcysteine positive control. CCK-8 and LDH would serve as co-primary injury readouts; apoptosis, mitochondrial membrane potential or reactive oxygen species, and dopaminergic phenotype endpoints would provide orthogonal confirmation.

Mechanistic follow-up would begin only after reproducible phenotypic protection is established. AKT–BCL2–CASP3 and MMP9 modules would then be assessed with pharmacological perturbation and orthogonal protein or activity readouts. Material-level work would separately quantify baicalein in the intended Polygonati Rhizoma preparation before any activity is attributed to that material. KCNH2 relations remain safety-only and require hERG-oriented assessment. The full prespecified matrix and completion status are retained in Supplementary Table S8 and data/planned_validation_matrix.csv; every entry remains prospective.

The study workflow and its explicit inference boundary are summarized in Figure 1.

## 3. Results

### 3.1. A Locked Ten-Plant Feasibility Panel Yielded 190 Plant-Disease Intersected Targets

The ten-plant panel was locked using regulatory and data feasibility criteria before disease targets were introduced. The analysis therefore describes what this panel yields under the stated data sources; it does not rank the ten plants against the entire official medicine–food homology pool. Non-panel materials remain outside the inference boundary.

**Figure 2:**
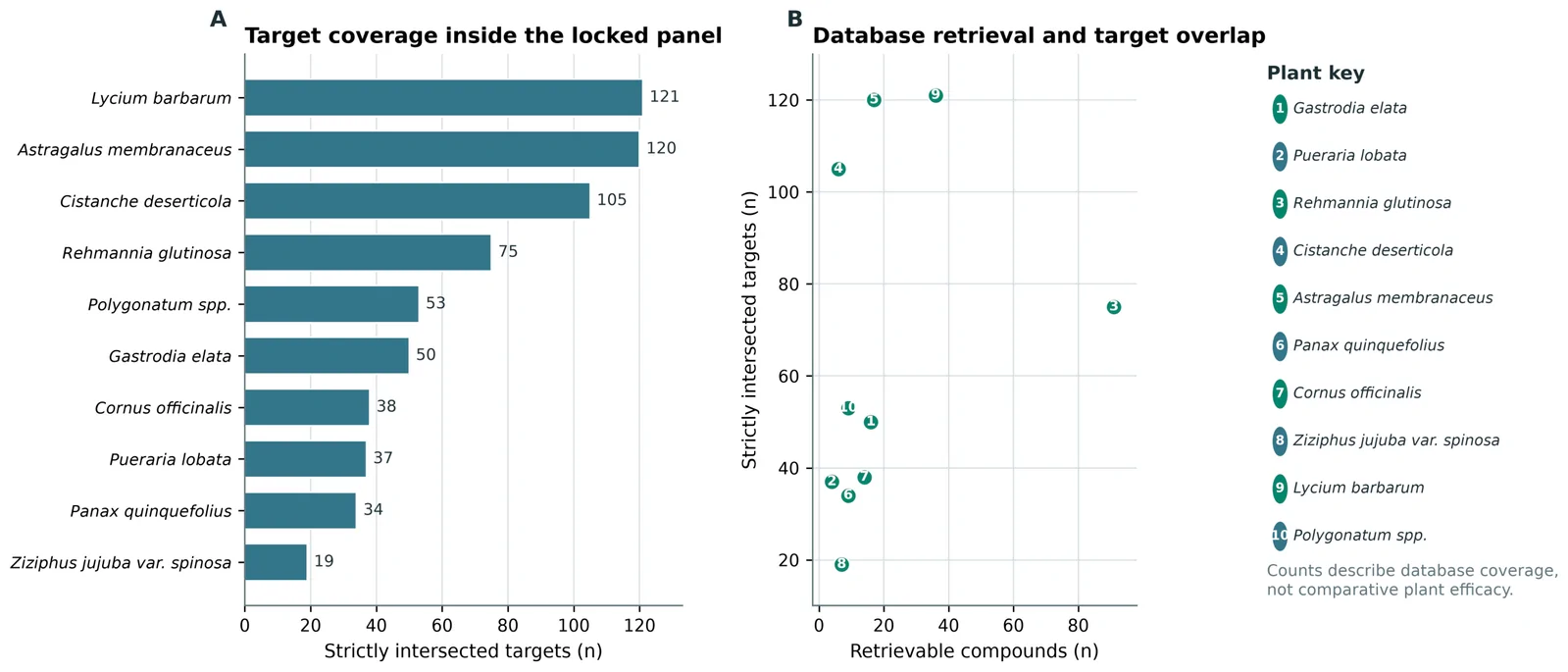
Database coverage within the locked ten-plant feasibility panel. Panel (**A**) reports strictly intersected target counts. Panel (**B**) relates retrievable compound counts to target overlap using numbered plant labels. Scientific names are given in the plant key. Counts describe database coverage and cannot be interpreted as comparative plant efficacy or superiority over non-panel materials.

Across the ten plants, 2346 compound–target records involved 382 unique human-mappable targets. Open Targets yielded 1237 unique PD-associated genes at a score of ≥0.1, and the retained CTD/Harmonizome branch yielded 517 at a score of ≥1.0; their deduplicated union contained 1631 genes. The unfiltered plant–disease intersection contained 192 genes. Strict plant-side mapping removed GABRA6 and NOS3 because both were represented only by mapped_candidate target name conversions, leaving 190 targets. This strict overlap represented 11.65% of the disease union and 49.74% of plant targets and defined the denominator for PPI, enrichment and expression-context analyses. As emphasized in Figure 2, plant-level target counts reflect database retrievability within the locked panel, not comparative efficacy, material quality or superiority over non-panel materials.

### 3.2. Post Hoc Robustness Audits Distinguished Single-Plant Stability from Database Dependence

Leaving out one plant at a time retained 173–190 of the 190 strict targets (91.05–100%). Exclusion of the dried Rehmanniae Radix working record had the largest effect, retaining 173 targets and removing 17. Omission of Polygonati Rhizoma retained 183 (96.32%), and omission of each of the other eight plants retained 186–190 (97.89–100%). Thus, the majority of the strict intersection was not determined by any single retained plant. This within-panel result does not test replacement by non-panel materials or representativeness of the full official source pool (Supplementary Table S9).

Disease-target records were more source-dependent. Of the 190 strict targets, 49 were supported by both source branches, 47 by Open Targets only and 94 by CTD/Harmonizome only. Open Targets alone retained 96 targets (50.53%), whereas CTD/Harmonizome alone retained 143 (75.26%). Among the five tracked core/context targets, the Open Targets-only branch retained AKT1 but not MMP9, BCL2, CASP3 or KCNH2, whereas the CTD/Harmonizome-only branch retained all five. Tightening the Open Targets cutoff to 0.2 with CTD/Harmonizome fixed at ≥1.0 retained 177 (93.16%); tightening CTD/Harmonizome to ≥1.5 with Open Targets fixed at ≥0.1 retained 140 (73.68%); applying both changes retained 125 (65.79%). Under the more stringent joint rule of Open Targets ≥ 0.3 or CTD/Harmonizome ≥ 2.0, AKT1, BCL2 and CASP3 remained in the strict set, whereas MMP9 and KCNH2 did not. KCNH2 remained safety-only under every decision rule. These results expose curation and threshold dependence rather than validating one preferred cutoff (Supplementary Table S9).

The rule ablation audit identified four concrete interpretation failures. A PPI-centrality-only reading would place common hubs such as GAPDH, TNF, IL6 and TP53 alongside follow-up targets without establishing PD specificity. A docking-only reading would favor quercetin-MMP9 (−10.383 kcal/mol) over baicalein-MMP9 (−9.701 kcal/mol) on pose score alone. A whole-blood-feature-only reading would elevate KCNH2, and a source-blind reading would conceal baicalein’s material-provenance gap. The complete rules instead retained quercetin-MMP9 as a liability-tagged comparator, KCNH2 as safety-only and baicalein pairs as source-pending, caution-tagged follow-up records. This audit demonstrates that the safeguards change interpretation; without external outcome labels, it does not demonstrate superior predictive accuracy (Supplementary Table S10).

### 3.3. Plant–Compound Provenance Mapping Clarified Candidate–Source Relationships

**Figure 3:**
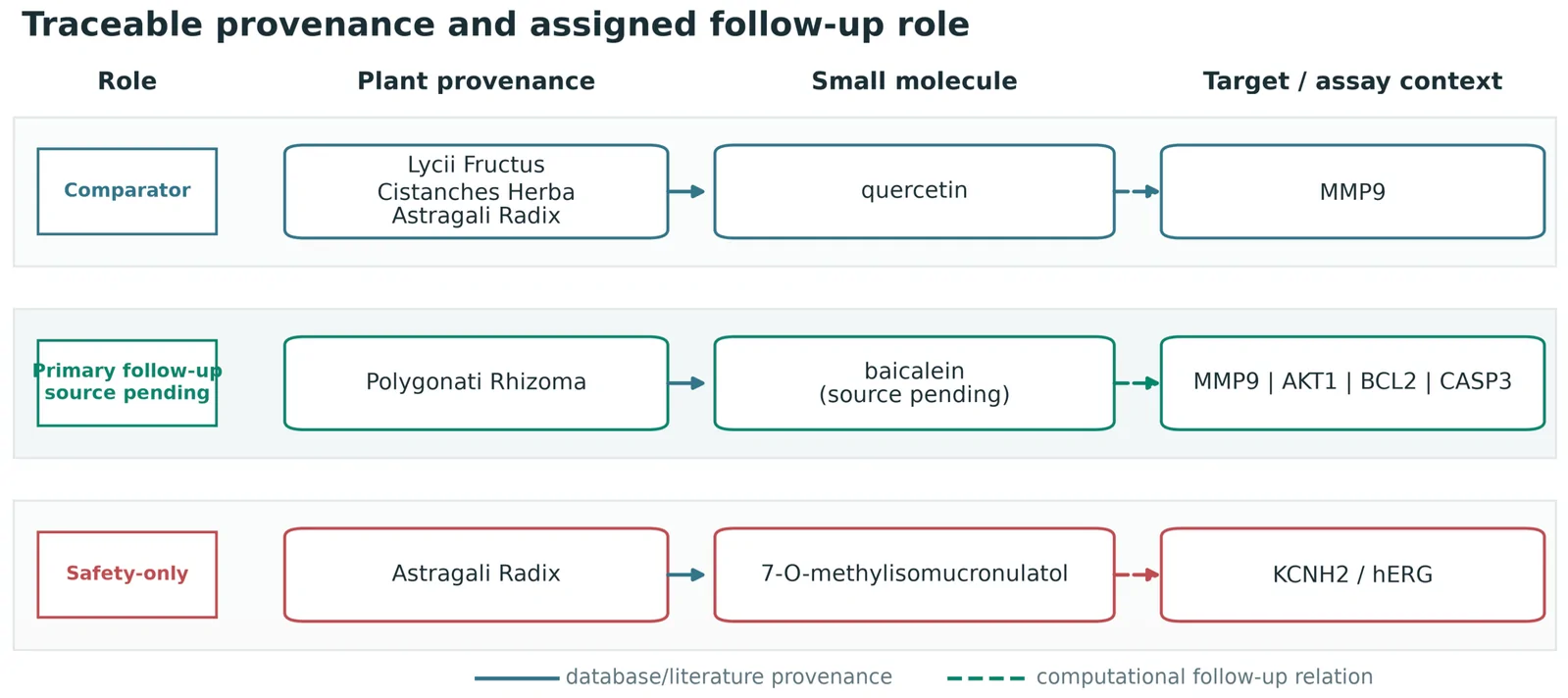
Traceable plant–compound–target provenance shown as three non-overlapping role lanes. Blue, green, and red denote the comparator, primary follow-up/source-pending, and safety-only role lanes, respectively. Solid plant-to-compound arrows indicate database or literature provenance, not measured material content. Dashed compound-to-target arrows indicate computational follow-up relations, not binding or direction of effect. Baicalein remains source-pending, and 7-O-methylisomucronulatol-KCNH2/hERG remains safety-only. Abbreviations: KCNH2, potassium voltage-gated channel subfamily H member 2; hERG, human ether-à-go-go-related gene.

The provenance map separated multi-plant database records, source-pending relations and safety-only signals before target interpretation. Quercetin was linked to Lycii Fructus, Cistanches Herba and Astragali Radix and was retained as a broad comparator. Baicalein remained limited to the Polygonati Rhizoma database/literature provenance layer and required material confirmation. The 7-O-methylisomucronulatol-KCNH2 relation was retained only to expose hERG liability. These statuses prevent a database relation from being rewritten as measured phytochemistry (Figure 3).

### 3.4. PPI Topology and Enrichment Exposed Broad, Non-Specific Biological Context

**Figure 4:**
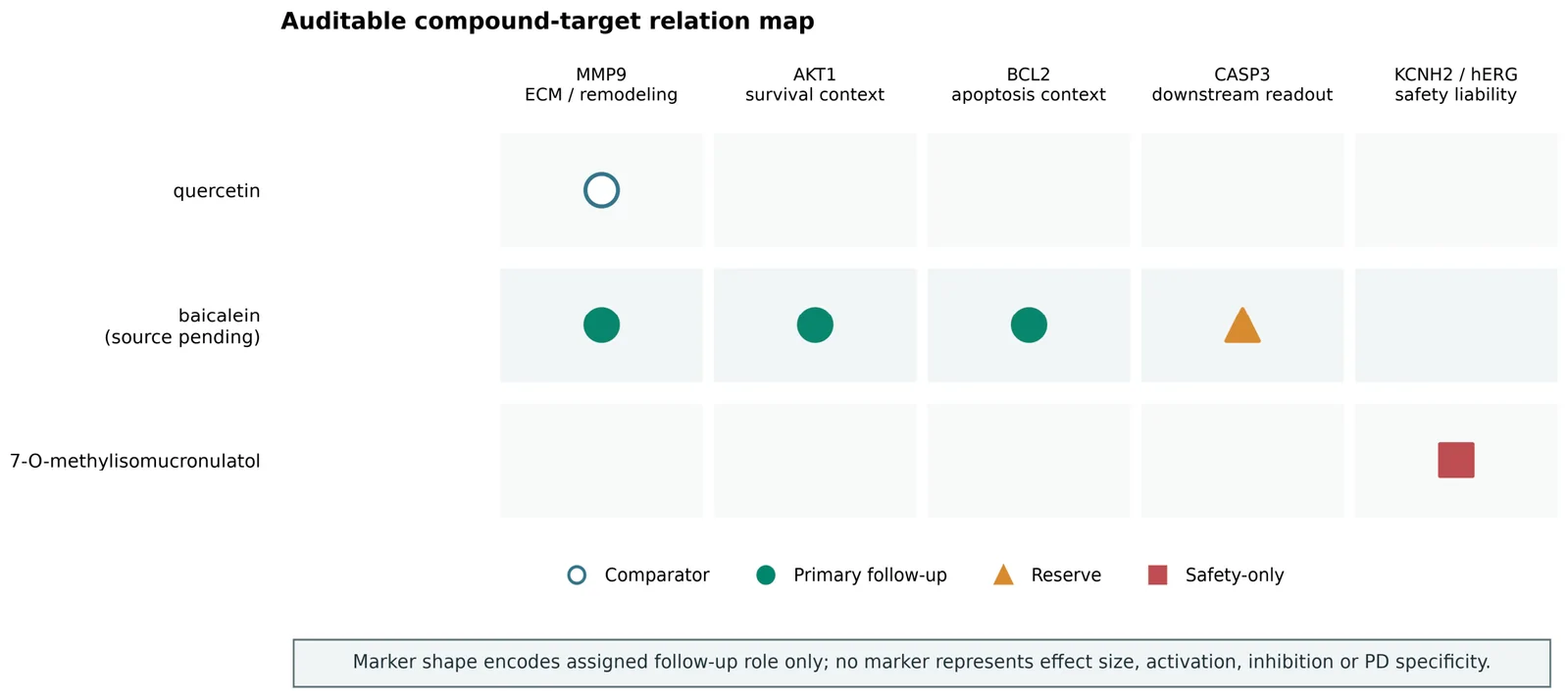
Auditable relation map for the retained compound–target set. Marker shape denotes categorical follow-up role: comparator, primary follow-up, reserve or safety-only. Target columns state broad assay contexts rather than causal mechanisms. The display contains no additive score, direction-of-effect claim or PD-specificity claim.

**Figure 5:**
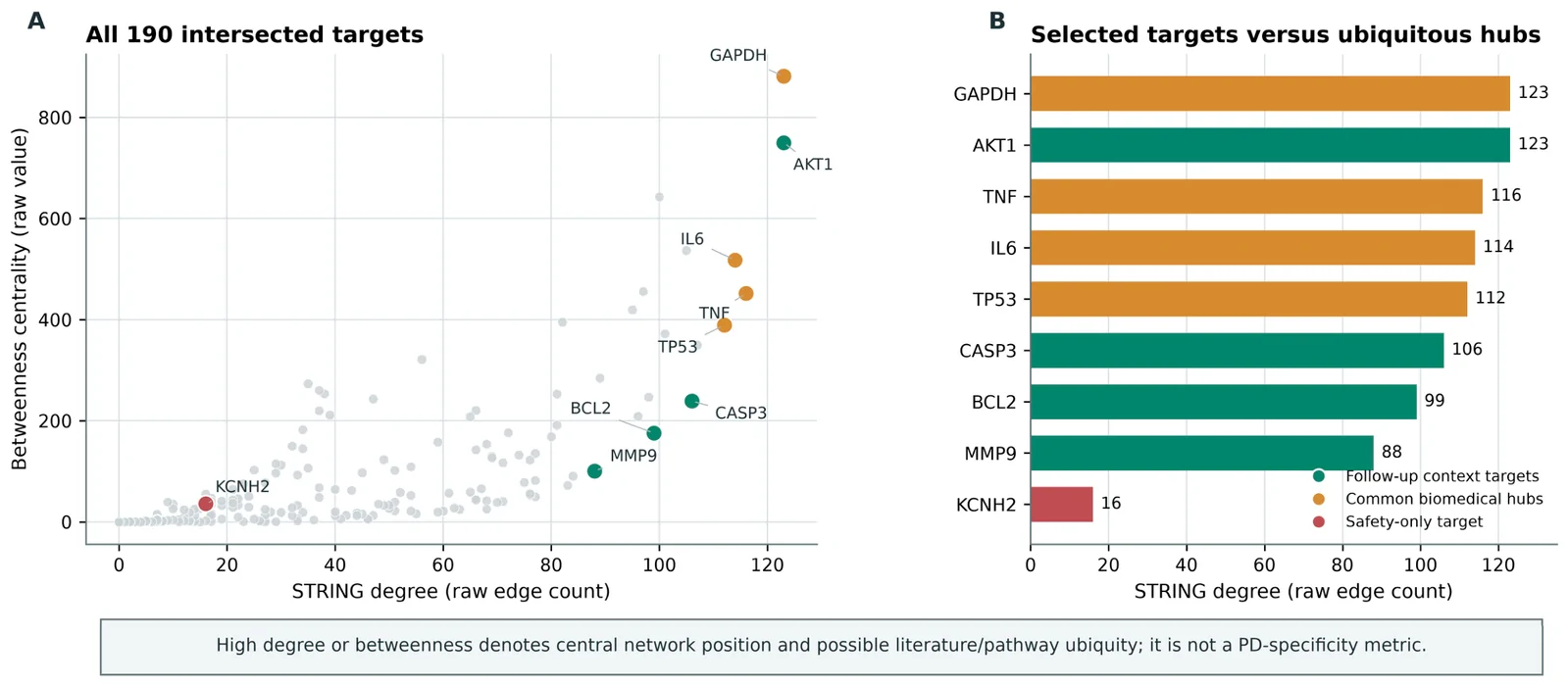
PPI ubiquity audit of the 190 intersected targets. Panel (**A**) plots raw STRING degree against raw betweenness; light-gray points represent the remaining intersected targets that were not individually labeled. Panel (**B**) reports raw degree for selected follow-up-context targets, common biomedical hubs and the safety-only KCNH2 target. Centrality is interpreted as network position and possible literature or pathway ubiquity, not PD specificity or independent mechanism evidence. Abbreviations: PPI, protein–protein interaction; STRING, Search Tool for the Retrieval of Interacting Genes/Proteins; KCNH2, potassium voltage-gated channel subfamily H member 2; PD, Parkinson’s disease.

In the STRING network, 187 of the 190 targets entered the largest interaction component, and 3800 filtered edges were retained. Raw degree placed AKT1 (123), CASP3 (106), BCL2 (99) and MMP9 (88) among well-connected nodes, but GAPDH also had a degree of 123, while TNF, IL6 and TP53 had degrees of 116, 114 and 112. This distribution shows that high centrality overlaps strongly with common biomedical hubs. The revised PPI display therefore uses raw degree and betweenness only to audit network ubiquity; no composite centrality score is used, and centrality is not treated as PD specificity. Figure 4 separately displays the retained compound–target follow-up roles, while Figure 5 compares candidate-context targets with common hubs.

**Figure 6:**
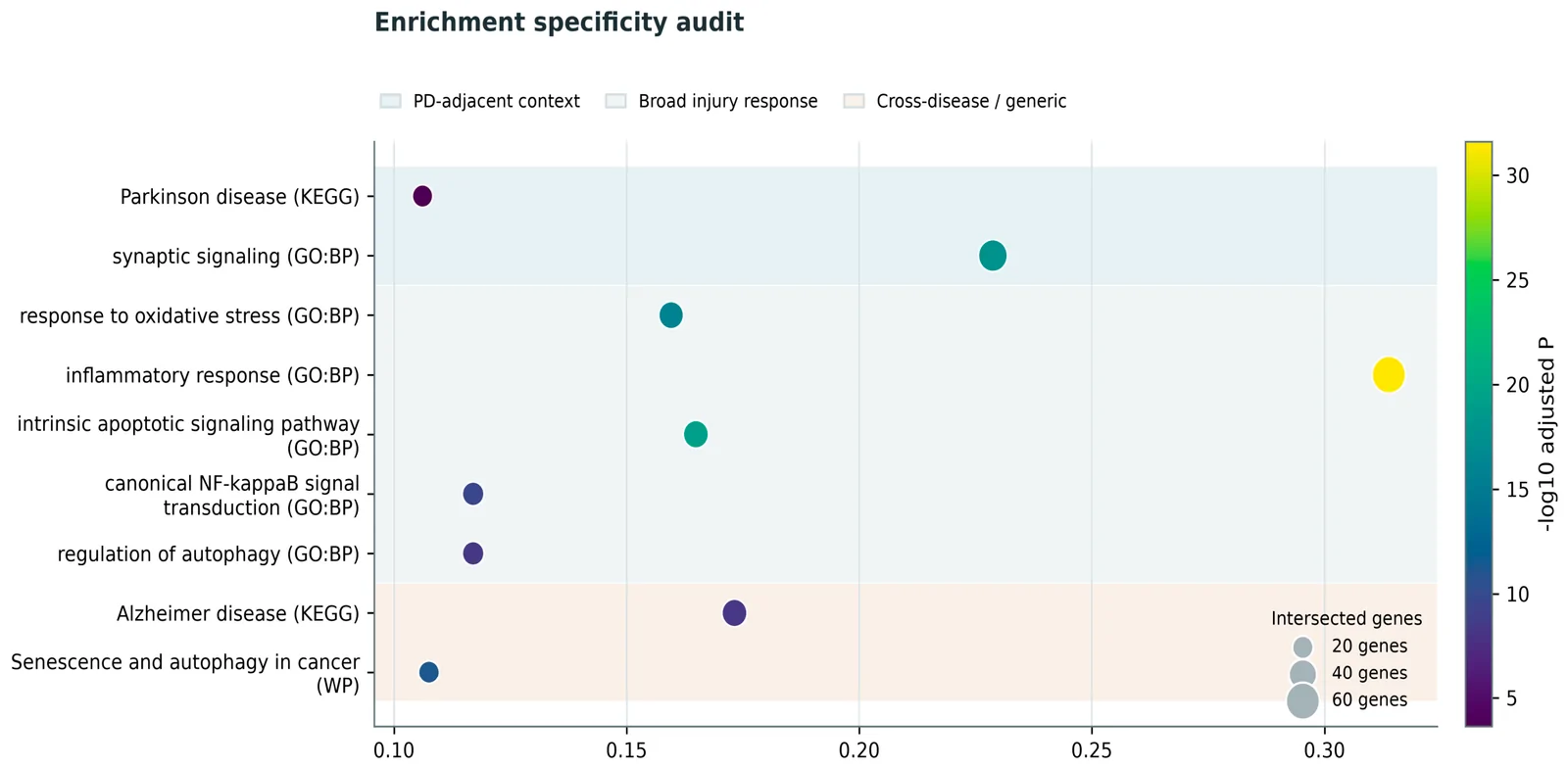
Specificity audit of representative enrichment terms. Gene ratios are shown on the x-axis, bubble size denotes the number of intersected genes and color denotes −log10 adjusted P. Terms are grouped as PD-adjacent context, broad injury response or cross-disease/generic context. Broad and cross-disease terms are retained deliberately to show the specificity limitation; this is not a pathway rank or evidence of a PD-specific mechanism.

Representative enrichment results included synaptic signaling and the KEGG Parkinson disease term, but they co-occurred with broad injury response processes, such as oxidative stress, inflammatory response, apoptosis, NF-kappaB signaling and autophagy, as well as cross-disease annotations for Alzheimer disease and cancer-associated senescence/autophagy. This mixture is evidence of biological breadth rather than pathway specificity. Figure 6 therefore groups representative terms into PD-adjacent, broad injury response and cross-disease or generic context; it does not rank mechanisms or establish target engagement.

### 3.5. Transcriptomic Evidence and Single-Nucleus Localization Provided Target Context

**Figure 7:**
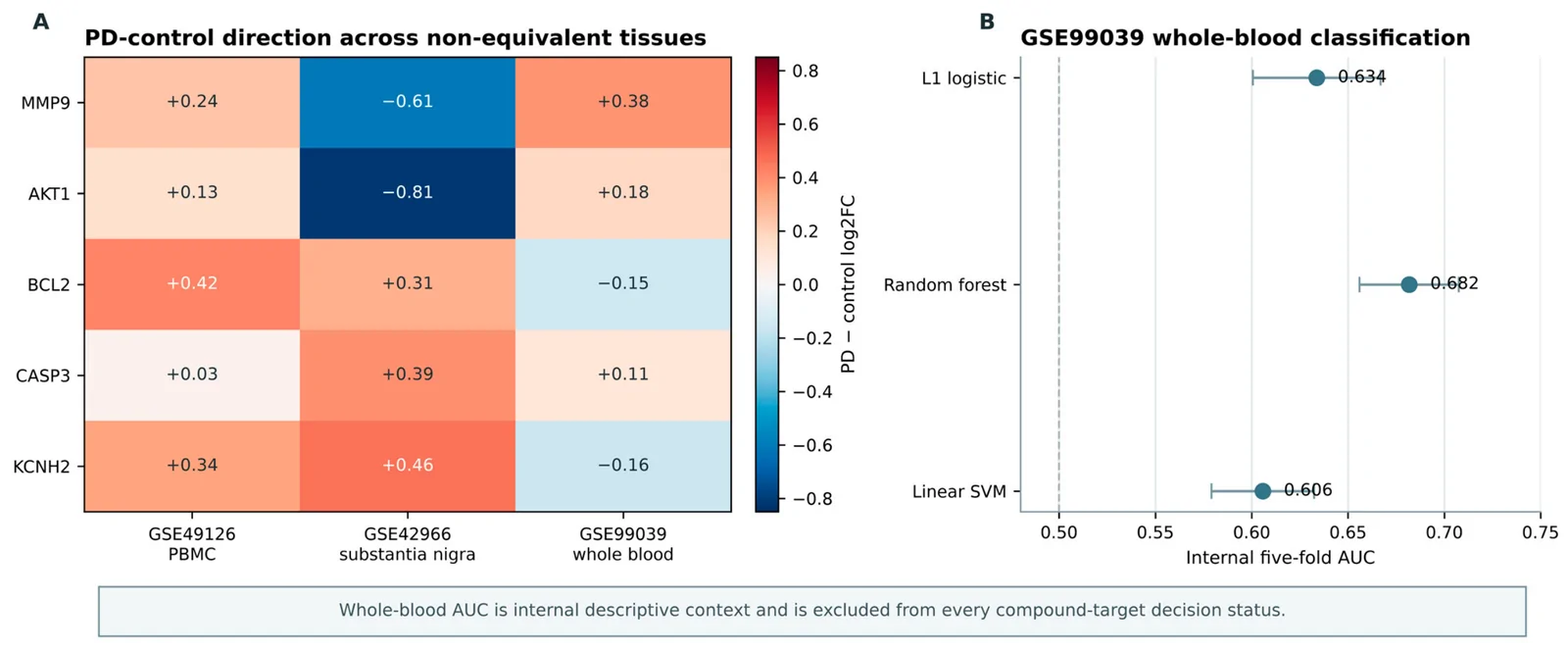
Tissue-dependent expression context and the boundary of whole-blood classification. Panel (**A**) shows PD-versus-control log2FC for five target-context genes across PBMC, substantia nigra and whole blood. Panel (**B**) reports internal five-fold AUCs in GSE99039; error bars denote SD across five validation folds, not external uncertainty. The vertical dashed line indicates the chance-level reference (AUC = 0.50).Whole-blood models are descriptive peripheral context, are excluded from candidate status and do not establish a substantia nigra mechanism or diagnostic model. Abbreviations: PD, Parkinson’s disease; log2FC, log2 fold change; PBMC, peripheral blood mononuclear cell; AUC, area under the receiver operating characteristic curve; SD, standard deviation.

Expression direction varied across PBMC, substantia nigra and whole blood, underscoring tissue heterogeneity. In GSE99039, random forest achieved a five-fold cross-validated AUC of 0.682 (SD 0.026; fold range 0.654–0.706; descriptive 95% CI 0.650–0.714), while L1-logistic regression achieved 0.634 (SD 0.033; range 0.592–0.670; 95% CI 0.593–0.675) and linear SVM achieved 0.606 (SD 0.027; range 0.574–0.643; 95% CI 0.573–0.639). These intervals summarize variation across five internal folds and are not external validation uncertainty. Random-forest importance placed KCNH2, neutrophil cytosolic factor 1 (NCF1) and MMP9 among its leading records, while L1 coefficients placed ERBB2 and KCNH2 near the top. The prominence of the cardiac ion-channel safety gene KCNH2 and the neutrophil NADPH-oxidase component NCF1 could reflect peripheral blood cell composition or model noise rather than neuronal target relevance. Because feature selection was not nested and whole blood is not substantia nigra, these rankings were treated as descriptive peripheral records, not mechanisms, biomarkers or compound–target evidence. KCNH2/hERG remained safety-only (Figure 7).

The strict-target GSE99039 matrix contained several dopamine- or monoamine-related genes, including SLC6A3, DRD1, DRD2, DRD4, DRD5, MAOA, MAOB, SLC6A2, SLC6A4 and TAAR1. These markers helped keep the interpretation anchored to Parkinson’s disease biology, while canonical markers absent from the strict matrix, such as TH, SLC18A2 and DDC, were retained as phenotype-context references rather than forced into the computational target ranking.

**Figure 8:**
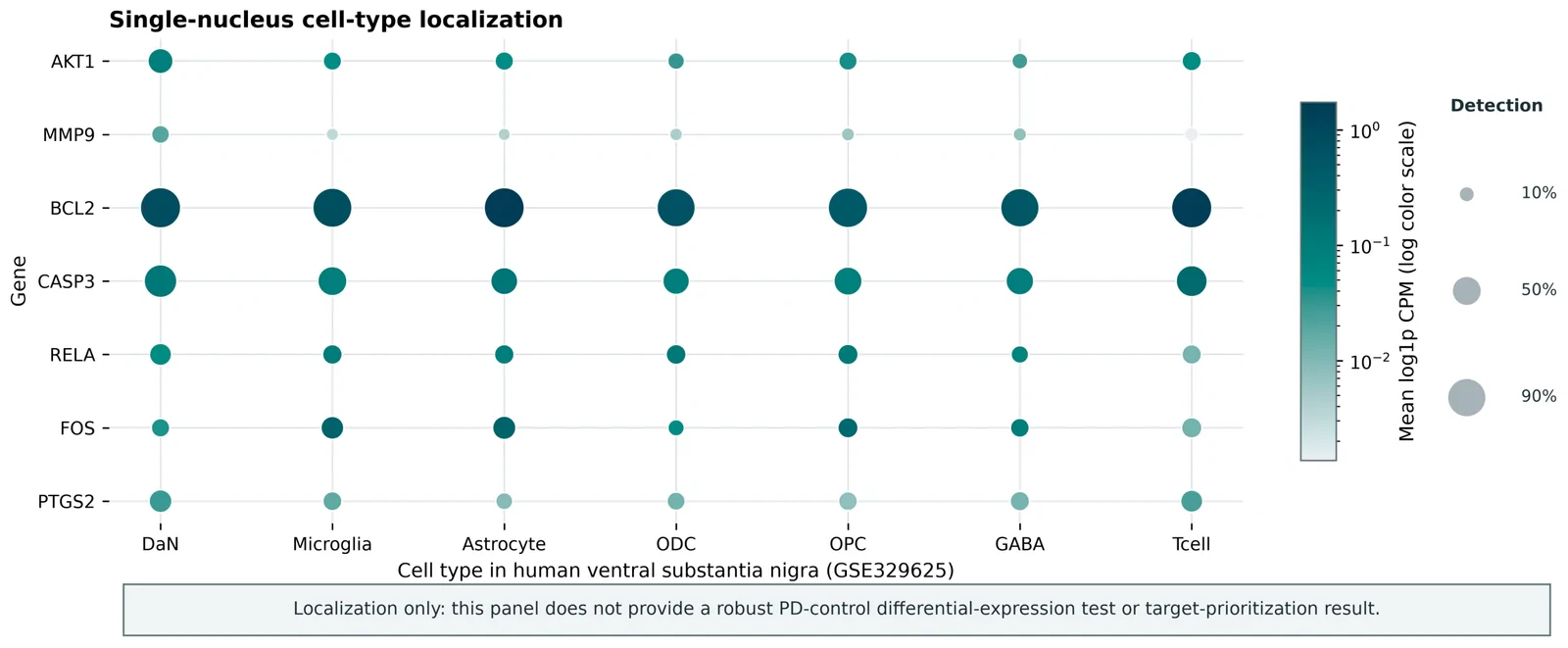
Cell-type localization of candidate and context genes in human substantia nigra single-nucleus data. Dot size indicates the proportion of nuclei with detected expression, and color indicates mean per-nucleus log1p expression after 10,000-count scaling. Localization includes CTR, PD and ILBD nuclei. The plot is not a PD-versus-control differential expression result or prioritization layer. Abbreviations: CTR, control; PD, Parkinson’s disease; ILBD, incidental Lewy body disease; DaN, dopaminergic neuron; ODC, oligodendrocyte; OPC, oligodendrocyte precursor cell.

Single-nucleus data from human substantia nigra supported cell-type localization and a limited exploratory donor-level comparison. In the author-annotated dopaminergic-neuron population, BCL2 was detected in 97.71% of nuclei, CASP3 in 62.64%, AKT1 in 34.56% and MMP9 in 14.95%. After count aggregation within donor and cell type, analyzable dopaminergic neuron summaries were available from 7 CTR and 8 PD donors; ILBD donors were excluded from the disease comparison. AKT1 showed an unadjusted difference (PD-CTR = −0.891 log1p CPM; Welch *p* = 0.024), but this did not remain significant after correction within the dopaminergic neuron tests (FDR = 0.465) or across all tested gene–cell-type combinations (global FDR = 0.779). MMP9, BCL2 and CASP3 were also non-significant, and no tested gene–cell-type combination reached FDR < 0.05. The donor-level estimates were therefore uninformative at the available sample size. They were retained only as underpowered background and were not interpreted as disease-state validation, negative biological evidence or a candidate decision layer. Figure 8 shows localization, and Table 4 reports the four prespecified dopaminergic neuron comparisons.

Values were calculated after summing raw gene counts and total counts within each donor’s annotated dopaminergic neurons and transforming as log1p (counts per million). The comparison used 7 CTR and 8 PD donors; ILBD donors were excluded. Welch *p* values are unadjusted, and the displayed FDR values are Benjamini–Hochberg corrections within the dopaminergic neuron gene set. No displayed comparison reached FDR < 0.05. Estimates were uninformative at the available sample size and were not used for target prioritization.

### 3.6. Evidence Domain Assessment Separated Follow-Up, Comparator, Reserve and Safety-Only Pairs

The retained table contained 31 compound–target records, each reported by source, PD-context, structural and liability domains in Supplementary Table S5. Quercetin-MMP9 had a complete MMP9 docking/ProLIF record but was assigned as a comparator because quercetin is a common network pharmacology component and carried unfavorable BBB proxy and PAINS/Brenk flags. Baicalein-MMP9, baicalein-AKT1 and baicalein-BCL2 met the computational primary follow-up rule but remained caution-tagged because the Polygonati Rhizoma source relation is not material-confirmed and baicalein carried PAINS/Brenk alerts. Baicalein-CASP3 was a downstream reserve readout, and 7-O-methylisomucronulatol-KCNH2 was safety-only (Table 5).

Decision status followed the deterministic rules in Table 3. Presence in one domain could not compensate numerically for a missing core domain, and liabilities could only maintain or downgrade a status. The labels describe follow-up priority inside this computational dataset; they are not probabilities, effect sizes or efficacy ranks.

The full 31-pair matrix is retained to show missing structural benchmarks and negative or safety-oriented classifications, thereby avoiding selective presentation of only favorable pairs.

**Figure 9:**
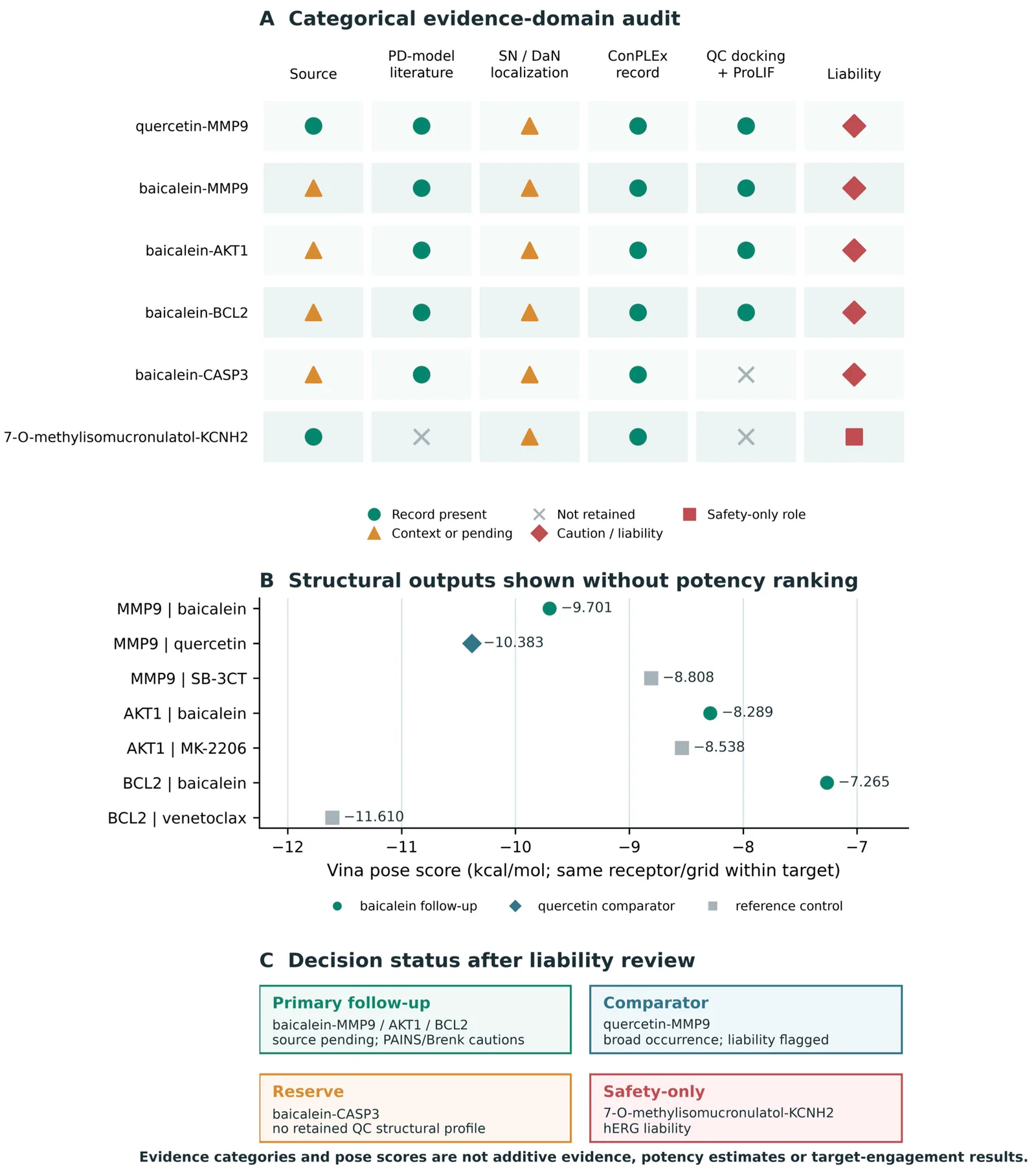
Categorical evidence domain and structural audit. Panel (**A**) uses marker shapes, rather than numerical codes, to distinguish a present record, context or pending status, a non-retained record, a caution/liability and a safety-only role. Panel (**B**) reports same-grid Vina pose scores for follow-up compounds, the quercetin comparator and target-oriented reference controls without implying potency ranking. Panel (**C**) retains the categorical decision status and mandatory cautions. No panel indicates efficacy or target engagement. Abbreviations: PD, Parkinson’s disease; SN, substantia nigra; DaN, dopaminergic neuron; QC, quality control; ProLIF, protein–ligand interaction fingerprint; MMP9, matrix metallopeptidase 9; AKT1, AKT serine/threonine kinase 1; BCL2, BCL2 apoptosis regulator; CASP3, caspase 3; KCNH2, potassium voltage-gated channel subfamily H member 2; hERG, human ether-à-go-go-related gene; PAINS, pan-assay interference compounds.

ConPLex produced continuous auxiliary scores for 30 of 31 pairs with complete reviewed human sequence-SMILES input. Isorhamnetin-NCF1 had no retained score because a reviewed sequence mapping was unavailable. No protein structure coordinate was supplied to ConPLex, and no binary score threshold was applied. The separate same-grid docking audit used MMP9 2OVX/4MR, AKT1 3MV5/XFE and BCL2 4IEH/1E9. Quercetin-MMP9 gave −10.383 kcal/mol and 16 ProLIF events across nine residues. Baicalein-MMP9, baicalein-AKT1 and baicalein-BCL2 gave −9.701, −8.289 and −7.265 kcal/mol, with 18/7, 15/7 and 10/4 ProLIF events/unique residues. The corresponding controls gave −8.808, −8.538 and −11.610 kcal/mol. Candidate–control differences were not interpreted as potency because Vina energies are pose-scoring outputs. These results are summarized in Table 6 and Figure 9.

Vina affinity is reported in kcal/mol. Redocking RMSD, grid coordinates and run settings are given in Supplementary Table S6. ProLIF event and unique-residue counts use one standardized interaction fingerprint definition; ordinary distance-contact counts are not mixed into this table. The BCL2 result indicates BH3-groove pocket compatibility only.

## 4. Discussion

This study addresses a methodological problem in network pharmacology: broad disease-target overlap and enrichment can make common injury response nodes appear more specific than they are. The revised workflow therefore separates a locked source panel, denominator-controlled target overlap, tissue context, structural quality control and liabilities. Its contribution is an auditable downgrade process, not a claim that MMP9, AKT1, BCL2 or CASP3 are novel or PD-specific targets.

The prioritized target axis gains disease-context coherence when interpreted against established Parkinson’s disease mechanisms, but the axis should be read as a broad injury response hypothesis rather than a biomarker panel unique to Parkinson’s disease. AKT signaling is commonly associated with neuronal survival and natural-product-mediated neuroprotection [42]. BCL2 links mitochondrial apoptosis, autophagy balance and dopaminergic neuronal vulnerability [43]. MMP9 is connected to neuroinflammation, blood–brain barrier dysfunction and extracellular matrix remodeling in Parkinson’s disease-related models and clinical studies [44,45,46,47]. These pathways are relevant to Parkinson’s disease models but are not unique to Parkinson’s disease; therefore, they support a follow-up testing route rather than proving an exclusive Parkinson’s disease mechanism by themselves. Dopamine/monoamine marker coverage and substantia nigra single-nucleus localization further placed these targets within a disease-relevant cell-type background.

Classical Parkinson’s disease mechanisms provide an additional frame for this interpretation. SNCA-, LRRK2-, PRKN/PINK1-, PARK7-, GBA- and MAPT-related pathways point to protein aggregation, mitochondrial dysfunction, lysosomal impairment, oxidative stress and neuroinflammation. Within this context, AKT1, MMP9 and BCL2 can be viewed as downstream or response nodes through which plant-derived compounds may influence survival, inflammatory remodeling and apoptosis-related processes. This positioning gives the target set a clearer Parkinson’s disease context than a generic hub-gene ranking alone.

The provenance map constrains structural interpretation. Quercetin-MMP9 has a complete pose-level record but is retained as a comparator because broad occurrence and chemical liability flags reduce specificity. Baicalein-MMP9, baicalein-AKT1 and baicalein-BCL2 meet the structural follow-up rule, yet their source remains material-unconfirmed. Thus, structural completeness cannot repair a provenance gap.

Baicalein should be read as a contextually plausible but liability-flagged candidate signal, not as a confirmed medicine–food homology marker compound. Prior studies have reported protection against MPP^+^/MPTP-induced injury, rotenone-induced SH-SY5Y apoptosis, 6-OHDA-induced toxicity and microglial inflammatory activation [48,49,50,51,52,53,54]. However, its plant–compound provenance in this project remains at the TCMSP Polygonati Rhizoma record and Polygonatum cyrtonema metabolite-table level, which justifies retention for computational comparison but not a claim of quantified content or contribution in a future experimental material [24]. The newly checked Chinese dissertation on Polygonatum sibiricum supports the broader chemical diversity of Polygonatum rhizomes, including steroidal saponins and homoisoflavones, but it does not confirm baicalein as a measured constituent [25]. ConPLex, docking and ProLIF results add structural prioritization evidence for baicalein-AKT1, baicalein-MMP9 and baicalein-BCL2, whereas PAINS/Brenk alerts require counter-screening and orthogonal assays before any biochemical or cellular target engagement claim.

Quercetin-MMP9 illustrates why a complete computational record is not equivalent to a preferred therapeutic lead. Quercetin is frequent in network pharmacology datasets, had an unfavorable BBB-oriented proxy and triggered PAINS/Brenk alerts. Its MMP9 docking and ProLIF results make it useful as a structural comparator and stress test for the framework, while future work would still require counter-screening and direct target-engagement assays.

Published natural-product PD studies commonly combine network pharmacology, enrichment and docking, sometimes with transcriptomic or animal validation [55,56,57]. The present route differs by locking a disease-independent feasibility panel, reporting the 1631-to-190 denominator, excluding whole-blood ML from prioritization, applying same-grid controls and standardized ProLIF counts, and using non-weighted downgrade rules. The within-panel jackknife showed that no single retained plant determined most of the overlap, whereas branch deletion and threshold tightening showed substantial disease-target curation dependence. The rule-ablation audit also made the consequences of removing provenance, liability and tissue-role safeguards explicit. Together these analyses improve internal auditability, but they do not establish external predictive superiority or transfer to other plant panels, diseases or databases.

Plant-specific evidence also clarifies the scope of the claims. Defined Lycium barbarum glycopeptide and polysaccharide preparations have been tested in MPTP, 6-OHDA or related PD models [58,59]. A prior Astragali Radix study explicitly combined network pharmacology with experimental validation [60], whereas Polygonatum sibiricum polysaccharides have been studied in MPP^+^-based PD models through Akt/mTOR and Nrf2-related pathways [61]. The present framework differs from a conventional hub/pathway-to-validation route by requiring disclosed material provenance, tissue-role boundaries, structural quality control and liability-based downgrading before candidate status is assigned. Even so, these earlier studies do not validate the database-level baicalein-Polygonati Rhizoma relation, demonstrate baicalein content in the tested Polygonatum material or show that baicalein contributed to the reported polysaccharide effects.

A prespecified MPP^+^-treated SH-SY5Y route defines the next experimental decision gate rather than evidence already obtained. It separates cell identity and mode pilot checks from compound-only tolerability, CCK-8/LDH screening, orthogonal apoptosis/mitochondrial/dopaminergic-phenotype endpoints and AKT–BCL2–CASP3/MMP9 dependency tests. Any protection or mechanism claim would require independent biological replicates, assay interference controls, concordant orthogonal readouts and appropriate perturbation evidence. Material-level baicalein confirmation remains a separate prerequisite for attributing standard compound activity to Polygonati Rhizoma.

An evidence stage comparison is possible even though direct efficacy comparison is not. Levodopa-based therapy belongs to established symptomatic care [1,2], whereas the baicalein evidence cited here remains at cell and animal PD-model stages [48,49,50,51,52,53,54]. Coenzyme Q10 and inosine both progressed to phase III disease modification trials, but neither slowed clinical progression in the reported trial [62,63]. Supplementary Table S11 therefore compares evidence stage, model and outcome category rather than cross-study effect sizes. This places the baicalein hypothesis below human efficacy testing and shows why favorable computational or preclinical signals cannot be treated as translational efficacy. The ligands in Table 6 remain target-oriented docking controls, not therapeutic comparators.

Several limitations remain. First, the ten-plant panel is a feasibility panel. The within-panel jackknife does not resolve selection bias relative to non-panel official materials. Second, disease-target results were sensitive to curation source and threshold, and the post hoc stress tests lack external outcome labels. Third, database compound–target records remain affected by literature frequency and heterogeneous curation. Fourth, baicalein is source-pending; current source records do not establish its abundance or contribution in Polygonati Rhizoma [24,25,61]. Fifth, PBMC, substantia nigra and whole blood are biologically non-equivalent; GSE42966 is small, GSE99039 lacks nested/external validation and the GSE329625 donor analysis was underpowered. Sixth, MMP9, AKT1, BCL2 and CASP3 are broad injury response nodes rather than PD-specific biomarkers. Seventh, BBB, Lipinski, PAINS and Brenk outputs are in silico proxies, and no measured exposure or brain availability data were available. ConPLex, docking, controls and ProLIF cannot establish direct binding, potency or cellular target engagement. Eighth, no biological validation was performed in the present study. The proposed SH-SY5Y experiments are prospective and cannot establish neuroprotection, target engagement or mechanism until completed with independent biological replicates, assay interference controls, and orthogonal injury and phenotype endpoints.

## 5. Conclusions

This study provides an auditable computational triage framework for a locked medicine–food homology feasibility panel. Robustness and rule-ablation analyses expose where candidate interpretation depends on plant selection, disease-target curation, source provenance, tissue role and structural liabilities. Baicalein-MMP9, baicalein-AKT1 and baicalein-BCL2 remain caution-tagged computational follow-up pairs, quercetin-MMP9 is a comparator, baicalein-CASP3 is a reserve readout and KCNH2 pairs are safety-only. These pairs remain computational hypotheses rather than validated neuroprotective or mechanistic relationships. The next steps are prospective MPP^+^-treated SH-SY5Y testing with assay interference controls, independent biological replicates, orthogonal injury and phenotype readouts, and candidate-axis dependency assays, together with material-level baicalein confirmation and hERG-oriented safety assessment.

## Figures and Tables

**Table 1:** Two-stage construction of the ten-plant medicine–food homology analytical panel.

Stage	Inclusion Criterion	Operation	Interpretation Boundary
Stage 0: Official source boundary	Plant-derived records in the 2002 baseline and 2019, 2023 and 2024 additions.	Separate plant records from animal, mineral and unresolved material types.	Defines regulatory scope; does not rank plants.
Stage 1: Mapping feasibility	Clear botanical/material mapping; TCMSP/ETCM retrievability; PubChem/SMILES structure; human target mapping.	Reserve records with unresolved identity, sparse retrieval or non-standardizable structures.	Disease targets and PD literature are not used at this stage.
Stage 2: Panel lock	Record completeness and chemical diversity sufficient for network, descriptor and structural modules.	Lock the ten-plant feasibility panel before disease-target integration.	No exhaustive comparison or sensitivity analysis across the full official plant pool.

Abbreviations: PD, Parkinson’s disease; TCMSP, Traditional Chinese Medicine Systems Pharmacology Database and Analysis Platform; ETCM, Encyclopedia of Traditional Chinese Medicine; SMILES, simplified molecular-input line-entry system.

**Table 2:** Included medicine–food homology plants and database coverage.

Botanical Source	Part	Regulatory Source	Database Query	Compounds	Strict PD Targets
*Gastrodia elata Blume*	rhizome	NHC/SAMR 2023 No. 9	Gastrodiae Rhizoma; ETCM2.0	16	50
*Pueraria lobata (Willd.) Ohwi*	root	NHC 2002 list	Puerariae Lobatae Radix; TCMSP	4	37
*Rehmannia glutinosa (Gaertn.) DC.*	root; dried-root working record	NHC/SAMR 2024 No. 4	Rehmanniae Radix; ETCM2.0	91	75
*Cistanche deserticola Y.C.Ma*	succulent stem	NHC/SAMR 2023 No. 9	Cistanches Herba; TCMSP	6	105
*Astragalus membranaceus (Fisch.) Bunge/A. mongholicus Bunge*	root	NHC/SAMR 2023 No. 9	Astragali Radix; TCMSP	17	120
*Panax quinquefolius* L.	root	NHC/SAMR 2023 No. 9	Panacis Quinquefolii Radix; TCMSP	9	34
*Cornus officinalis Siebold & Zucc.*	fruit pulp	NHC/SAMR 2023 No. 9	Corni Fructus; TCMSP	14	38
*Ziziphus jujuba Mill. var. spinosa (Bunge) Hu ex H.F.Chow*	seed	NHC 2002 list	Ziziphi Spinosae Semen; TCMSP	7	19
*Lycium barbarum* L.	fruit	NHC 2002 list	Lycii Fructus; TCMSP	36	121
*Polygonatum sibiricum Redoute/P. cyrtonema Hua/P. kingianum Coll. et Hemsl.*	rhizome	NHC 2002 list	Polygonati Rhizoma; TCMSP	9	53

Abbreviations: NHC, National Health Commission; SAMR, State Administration for Market Regulation; PD, Parkinson’s disease; TCMSP, Traditional Chinese Medicine Systems Pharmacology Database and Analysis Platform; ETCM, Encyclopedia of Traditional Chinese Medicine.

**Table 3:** Deterministic rules for non-weighted evidence domain assignment.

Decision Component	Operational Rule	Effect on Status	Reason
Evidence domains	Report source traceability, PD context, structural support and developability/safety separately.	No additive score or letter grade.	Prevents heterogeneous evidence from being converted into an efficacy-like number.
Whole-blood ML	Report internal AUC and feature frequency descriptively; exclude from every pair decision.	Cannot promote or downgrade a pair.	Whole blood is not substantia nigra, and the model lacks nested/external validation.
Primary follow-up	Disclosed source status, at least one PD-context record and retained QC docking plus ProLIF.	Computational follow-up only; cautions remain visible.	Requires multiple non-identical domains without numerical compensation.
Comparator	Structurally complete but broad/common compound or major developability liabilities.	Used to test framework specificity.	A complete docking record is not sufficient for lead status.
Reserve	One or more core domains missing, including no retained QC structural benchmark.	Held for later evidence acquisition.	Missingness is shown rather than scored as zero.
Safety only	Pair centers on KCNH2/hERG or another off-target liability.	Excluded from PD mechanism claims.	Safety signals cannot be reframed as therapeutic targets.
Downgrade rule	Source, PAINS/Brenk structural alerts, BBB/lipophilicity or generalizability concerns can only maintain or lower status.	No liability can be offset by a stronger unrelated domain.	Makes the decision process deterministic and auditable.

Abbreviations: PD, Parkinson’s disease; ML, machine learning; AUC, area under the receiver operating characteristic curve; QC, quality control; ProLIF, protein–ligand interaction fingerprint; KCNH2, potassium voltage-gated channel subfamily H member 2; hERG, human ether-à-go-go-related gene; PAINS, pan-assay interference compounds; BBB, blood–brain barrier.

**Table 4:** Underpowered donor-level count-aggregated PD-versus-control comparison in dopaminergic neurons.

Gene	CTR Mean log1p CPM	PD Mean log1p CPM	PD–CTR	Welch P	DaN BH FDR
*AKT1*	3.078	2.187	−0.891	0.024	0.465
*MMP9*	1.325	1.361	0.036	0.923	0.968
*BCL2*	5.176	5.182	0.005	0.968	0.968
*CASP3*	2.857	2.909	0.052	0.630	0.854

Abbreviations: CTR, control; PD, Parkinson’s disease; log1p, natural logarithm of one plus the value; CPM, counts per million; DaN, dopaminergic neuron; BH, Benjamini–Hochberg; FDR, false discovery rate.

**Table 5:** Non-weighted evidence domain status for the main compound–target pairs.

Decision Status	Compound–Target	Source Status	PD-Context Domain	Structural Domain	Liabilities and Interpretation
Comparator	quercetin-MMP9	Multi-plant database record; no material quantification	Compound PD-model literature; MMP9 PD-context literature	2OVX QC docking; Vina −10.383; ProLIF 16 events/9 residues	Common network component; BBB proxy unfavorable; PAINS/Brenk structural alerts. Not a preferred efficacy lead.
Primary follow-up; caution-tagged	baicalein-MMP9	Polygonati Rhizoma relation; material confirmation pending	Baicalein PD-model literature; MMP9 PD-context literature	2OVX/4MR; RMSD 1.059 Å; Vina −9.701; ProLIF 18/7	PAINS/Brenk structural alerts; structural follow-up only.
Primary follow-up; caution-tagged	baicalein-AKT1	Polygonati Rhizoma relation; material confirmation pending	Baicalein PD-model literature; AKT survival context	3MV5/XFE; RMSD 0.348 Å; Vina −8.289; ProLIF 15/7	PAINS/Brenk structural alerts; no direct activation or engagement claim.
Primary follow-up; caution-tagged	baicalein-BCL2	Polygonati Rhizoma relation; material confirmation pending	Baicalein PD-model literature; apoptosis context	4IEH/1E9; RMSD 0.734 Å; Vina −7.265; ProLIF 10/4	PAINS/Brenk structural alerts; BH3-groove compatibility only.
Reserve	baicalein-CASP3	Polygonati Rhizoma relation; material confirmation pending	Downstream apoptosis readout	No retained QC docking/ProLIF profile	Not a primary direct-binding hypothesis.
Safety-only	7-O-methylisomucronulatol-KCNH2	Astragali Radix database record	No retained representative PD-model record	ConPLex record only; no retained QC docking/ProLIF	KCNH2/hERG liability; excluded from PD mechanism claims.

Abbreviations: PD, Parkinson’s disease; QC, quality control; RMSD, root mean square deviation; Å, ångström; ProLIF, protein–ligand interaction fingerprint; BBB, blood–brain barrier; PAINS, pan-assay interference compounds; KCNH2, potassium voltage-gated channel subfamily H member 2; hERG, human ether-à-go-go-related gene; BH3, BCL-2 homology 3.

**Table 6:** Molecular docking support for the main candidate pairs.

Compound–Target	PDB/Co-Ligand	Redocking/QC	Vina Score (kcal/mol)	ProLIF Events/Residues	Interpretation
quercetin-MMP9	2OVX/4MR	RMSD 1.059 Å	−10.383	16/9	Liability-tagged structural comparator.
baicalein-MMP9	2OVX/4MR	RMSD 1.059 Å	−9.701	18/7	Primary computational follow-up; no potency claim.
SB-3CT-MMP9	2OVX/4MR	Same prepared receptor/grid	−8.808	Not used	Reference-control context only.
baicalein-AKT1	3MV5/XFE	RMSD 0.348 Å	−8.289	15/7	Primary computational follow-up; no activation claim.
MK-2206-AKT1	3MV5/XFE	Same prepared receptor/grid	−8.538	Not used	Reference-control context only.
baicalein-BCL2	4IEH/1E9	RMSD 0.734 Å	−7.265	10/4	BH3-groove compatibility only.
venetoclax-BCL2	4IEH/1E9	Same prepared receptor/grid	−11.610	Not used	Reference-control context only.

Abbreviations: PDB, Protein Data Bank; QC, quality control; RMSD, root mean square deviation; Å, ångström; ProLIF, protein–ligand interaction fingerprint; BH3, BCL-2 homology 3. AutoDock Vina scores are reported in kcal/mol.

## Data Availability

Public transcriptomic datasets are available from the Gene Expression Omnibus (https://www.ncbi.nlm.nih.gov/geo/; accessed on 23 July 2026) under accession numbers GSE49126, GSE42966, GSE99039, and GSE329625. Derived computational records, scripts, parameters, and checksums are provided in MFH_PD_Computational_Reproducibility_Package.zip; Tables S1–S11 are provided in MFH_PD_Supplementary_Tables_Minor_Revision.xlsx. No new wet-laboratory data are reported in this article.

## Supplementary Materials

The following supporting information can be downloaded at https://www.mdpi.com/article/10.3390/biotech15030066/s1: Table S1: Official source status and scope record; Table S2: Dopaminergic phenotype-context definitions; Table S3: Reproducibility resources, accessions, thresholds, URLs, and package-relative records; Table S4: Dataset roles, heterogeneity, and analysis boundaries; Table S5: Full compound–target evidence domain matrix; Table S6: Docking controls, grids, preparation records, and quality control parameters; Table S7: Protein–ligand interaction fingerprint summaries; Table S8: Prespecified prospective cell validation matrix; Table S9: Plant panel and disease source robustness audits; Table S10: Decision-rule ablation audit; Table S11: Evidence-stage comparison of PD interventions and candidates. Files: MFH_PD_Supplementary_Tables_Minor_Revision.xlsx and MFH_PD_Computational_Reproducibility_Package.zip. No wet-laboratory data are included in either file.
